# Diseases Admitted as in and out Patients of the Physicians of the Westminster Hospital, between the 20th of February, and the 20th of March, 1799
*For the Account of Diseases in an Eastern District of London, see p. 215.


**Published:** 1799-04

**Authors:** 


### *Diſeaſes* admitted as in and out Patients of the Phyſicians of the Weſtminſter Hoſpital, between the 20th of February, and the 20th of March, 1799.*

| ACUTE DISEASES. | |
|---|---|
| Typhus | 4 |
| Synochus bilioſa | 1 |
| Phrenitis | 1 |
| Pneumonia | 2 |

| CHRONIC DISEASES. | |
|---|---|
| Amenorrhœa | 6 |
| Anaſarca | 3 |
| Aphthæ | 1 |
| Aſcites | 2 |
| Aſthenia | 3 |
| Aſthma | 2 |
| Catarrh | 8 |
| Convulſions | 1 |
| Cough | 4 |
| Diarrhœa | 6 |
| Dyſpepſia | 7 |
| Enterodynia | 3 |
| Epilepſy | 1 |
| Eryſipelas | 4 |
| Gonorrhagia | 1 |
| Hæmoptoe | 1 |
| Hæmorrhois | 2 |
| Hypochondriaſis | 1 |
| Hyſteria | 2 |
| Hydrocephalus | 1 |
| Itch | 5 |
| Iſchuria | 1 |
| Lepra Græcorum | 1 |
| Lichen | 1 |
| Lumbago | 1 |
| Menorrhagia | 1 |
| Ophthalmia | 1 |
| Paralyſis | 4 |
| Pleuriſy | 1 |
| Podagra | 1 |
| Rheumatiſm | 7 |
| Scurvy | 2 |
| Struma | 3 |
| Vomitio | 2 |
| Urticaria | 1 |
| Worms | 1 |

| PERIODIC DISEASES. | |
|---|---|
| Quotidian | 2 |
| Tertian | 1 |

Chronic

---

* For the Account of Diseases in an Eastern District of London, see p. 215.

Chronic rheumatifm is, perhaps, a more frequent difeafe in this hofpital than fome others, on account of the number of people employed in the gardens in the neighbourhood, who have feldom an opportunity of changing their clothes when wet.

The cafe of ifchuria above-mentioned was of the real kind, and proceeded from a fcirrhous enlargement of the vagina, preffing upon the ureters, at their entrance into the bladder. One ureter was enlarged to nearly an inch in diameter, and the pelvis of that kidney to the fize of an egg.

